# Extra-Adrenal Myelolipoma Presenting as Efferent Limb Obstruction

**DOI:** 10.1155/2012/718383

**Published:** 2012-07-25

**Authors:** Alexandria Conley, Elizabeth Klein, E. Edhayan, Richard Berri

**Affiliations:** Section of Surgical Oncology, Department of Surgery, Van Eslander Cancer Center, St. John Hospital and Medical Center, Detroit, MI 48236, USA

## Abstract

Myelolipomas are rare benign lesions composed of mature adipose tissue and immature hematopoetic cells. The adrenal gland is the most common location for these lesions, but cases of extra-adrenal myelolipomas have been described. The predominant location for extra-adrenal myelolipomas is the retroperitoneum, and very few reported cases describe these lesions in the peritoneal cavity. Typically these lesions are incidental findings and asymptomatic, but occasionally can present with symptoms secondary to mass effect. We present the case of a 72 year old man presenting with a gastric outlet obstruction secondary to an epigastric mass. The mass was resected and pathology was consistent with myelolipoma. This case illustrates an atypical location and presentation of a myelolipoma. These are rare tumors with only 5 intra-abdominal myelolipomas reported in the literature. This article is a review of the surgical literature and a discussion on myelolipomas. Knowledge of these rare entities can help ensure proper management of these patients, which may include early surgical intervention.

## 1. Introduction

Myelolipomas are benign lesions composed of mature adipose tissue and immature hematopoetic cells. The majority are found within the adrenal glands, but cases of extra-adrenal myelolipomas have been described. Extra-adrenal myelolipomas are commonly identified within the presacral area of the retroperitoneum [1], few cases describe these lesions within the peritoneal cavity. Typically these lesions are incidental findings and asymptomatic, but occasionally they may produce symptoms related to mass effect. We report the case of a seventy-two-year-old man with an intraperitoneal myelolipoma causing bowel obstruction.

## 2. Case History

### 2.1. Clinical Presentation

A 72-year-old man presented to the emergency department with one day of abdominal pain, distension, and vomiting. The patient reported that for the past five to ten years, he had occasional episodes of abdominal pain, distention, and food regurgitation for which he was hospitalized multiple times for suspected small bowel obstruction. Each of these episodes resolved spontaneously with nonoperative management. Workup during his most recent admission three years prior included a computed tomography scan of the abdomen that demonstrated a 6 cm × 10 cm nodular soft tissue mass in the midepigastrium. No surgical intervention was done at that time and the patient was lost to followup. His medical history was also significant for duodenal ulcers, Hepatitis B and C, alcoholic liver disease, and chronic pancreatitis. His past surgical history included Billroth II gastrojejunostomy, as well as remote inguinal herniorrhaphy and cholecystectomy.

On the day of presentation, the patient complained of abdominal pain, distention, and vomiting, but stated the pain was much more severe and unremitting than prior episodes. He had bilious emesis and was no longer passing flatus or having bowel movements. On physical examination, he was afebrile and his vital signs were normal. His abdomen was mildly distended, diffusely tender to palpation, and there were no peritoneal signs, palpable masses, or hernias.

### 2.2. Laboratory Studies and Diagnostic Imaging

**Figure 1:**
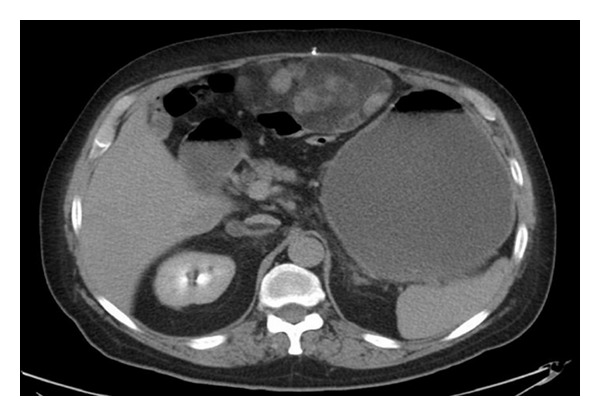
Computed tomographic axial image demonstrating anterior epigastric mass and dilated stomach and duodenum.

**Figure 2:**
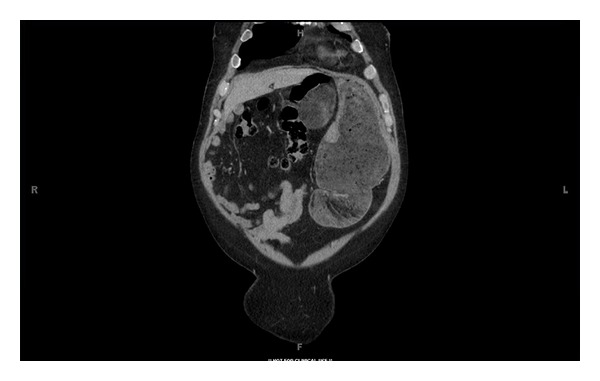
Computed tomographic coronal image showing epigastric mass abutting dilated stomach and duodenum.

**Figure 3:**
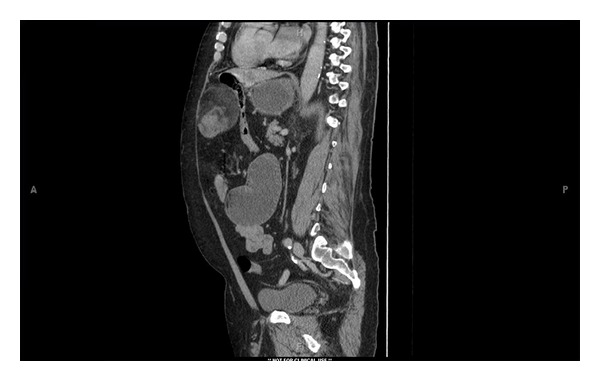
Computed tomographic sagittal image showing anterior epigastric mass compressing distal small bowel, causing dilated duodenum and stomach proximally.

Hematologic studies revealed a leukocytosis of 17,200/mm^3^. Abdominal radiograph showed a soft tissue density in the left midabdomen. Computed tomography scan of the abdomen demonstrated a 12.1 × 6.4 cm anterior epigastric lesion containing fat and soft tissue, as well as dilation of the stomach and duodenum (Figures 1–3).

## 3. Surgical Intervention and Postoperative ****Course

The patient was placed on bowel rest with intravenous fluids. A nasogastric tube was placed for decompression and returned a large amount of bilious output. Esophagogastroduodenoscopy demonstrated obstruction of the efferent limb of the gastrojejunostomy secondary to extrinsic compression. The afferent limb was patent with no obstruction or internal mass. The patient was taken to the operating room the following day for elective resection of the epigastric lesion.

**Figure 4:**
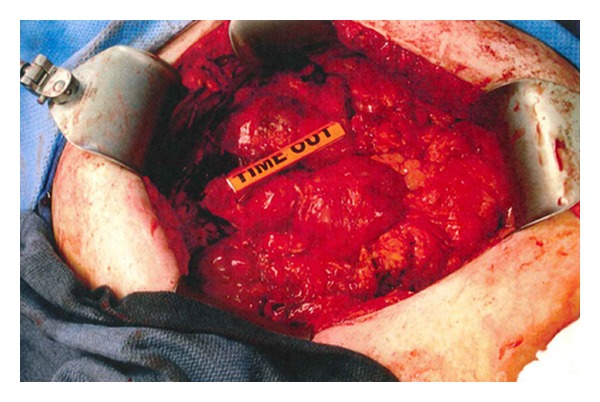
Intraoperative photo of intra-abdominal myelolipoma. Lesion is delineated by yellow ruler.

**Figure 5:**
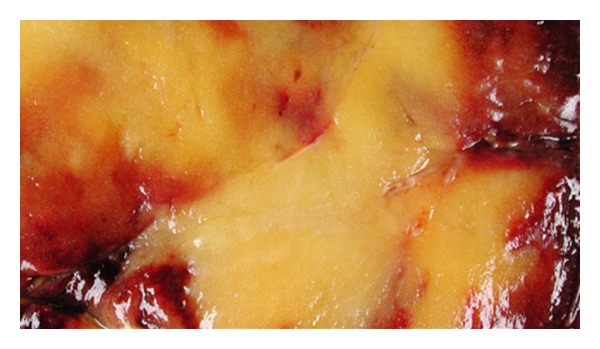
Cut surface of the resected mass: tan-brown to pale yellow color.

Intraoperatively, a midline incision was made and extensive lysis of adhesions was done. Once adequate mobilization of the intestines and surrounding omental tissue was complete, a 14 cm × 14 cm mass was visualized below the liver in the lesser omentum, extending superiorly to the liver, inferiorly to the transverse colon, laterally to the afferent limb of the gastrojejunostomy, and medially obstructing the efferent limb of the gastrojejunostomy (Figure 4). It was not adherent to the bowel, but was adherent to omentum that had been displaced toward the efferent and afferent limbs. The mass was dissected circumfrentially and resected en bloc with a portion of adhesed omentum. Frozen section diagnosis was consistent with a soft tissue sarcoma (Figure 5). The stomach was decompressed and the gastrojejunostomy was inspected from the afferent limb near the stomach towards the efferent limb and there was no obstruction at any point. The anastamosis was patent, so this was left intact with no revision of his Billroth II. The patient tolerated the procedure well and there were no complications. On postoperative day two he recovered bowel function and tolerated a regular diet. He was discharged home one week postoperatively with complete resolution of symptoms. Approximately 8 months after surgery he was seen in follow up and had no obstructive complaints.

## 4. Pathology

**Figure 6:**
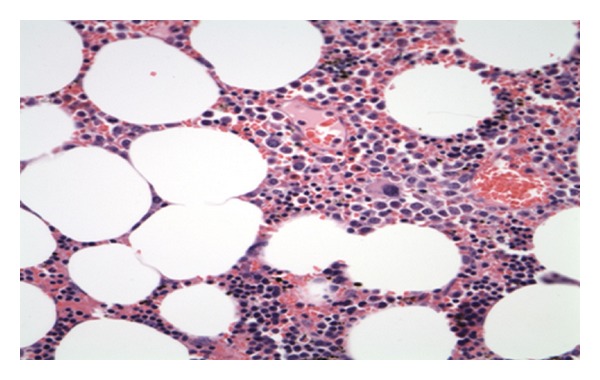
Microscopic examination of the specimen shows predominantly mature adipose tissue with scattered islands of trilinear hematopoietic cells including megakaryocytes. No atypia is identified. H&E stain 40×.

Final pathology demonstrated an irregular soft tissue mass (14.5 × 14.0 × 5.5 cm) with predominantly mature adipose tissue and scattered islands of trilinear hematopoietic cells, including megakaryocytes without atypia, consistent with a diagnosis of myelolipoma (Figure 6).

## 5. Discussion

Myelolipomas are rare, benign, nonfunctional lesions whose pathogenesis is uncertain. Proposed mechanisms include bone marrow emboli or embryonic nests of hematopoetic tissue [2] and metaplasia of reticuloendothelial cells in response to necrosis, infection, or stress [3]. They are usually found incidentally on imaging and occasionally during laparotomy or autopsy. Based on imaging alone, they are indistinguishable from other lipomatous tumors including liposarcomas or angiomyolipomas, so definitive diagnosis requires biopsy.

Generally, myelolipomas are asymptomatic and do not require intervention. However, they can sometimes present acutely with symptoms related to mass effect. Complications including abdominal pain, bleeding, constipation, urinary retention, and nausea/vomiting have all been described in the literature.

The predominant location for myelolipoma is the adrenal gland; however, extra-adrenal myelolipomas have been described. These lesions have been found in the liver, lung, mediastinum, mesentery, stomach, spleen, pelvis, and most commonly the retroperitoneal presacral region [4, 5]. Our review of the literature demonstrates there have been 45 reported cases of extra-adrenal myelolipomas, some with multiple lesions and locations including: 16 presacral, 9 retroperitoneal, 8 thoracic/mediastinal, 7 perirenal, 3 pelvic, 2 renal, 2 hepatic, 1 gastric, and 1 perivesicular [1, 6–23]. Based on this review and the lesion found in our patient, fewer than five cases of intraperitoneal myelolipomas have been described.

## 6. Conclusion

This case illustrates an atypical presentation and location of a myelolipoma and serves to make physicians aware of these variations. When an extra-adrenal myelolipoma is suspected on imaging, diagnostic procedures such as CT guided biopsy or fine needle aspiration can be used to confirm the diagnosis. However, in this case resection of the mass was essential to relieve the patient's bowel obstruction. The operation was successful in relieving the patient's symptoms as well as diagnosing and removing the tumor.
